# FluKB: A Knowledge-Based System for Influenza Vaccine Target Discovery and Analysis of the Immunological Properties of Influenza Viruses

**DOI:** 10.1155/2015/380975

**Published:** 2015-10-04

**Authors:** Christian Simon, Ulrich J. Kudahl, Jing Sun, Lars Rønn Olsen, Guang Lan Zhang, Ellis L. Reinherz, Vladimir Brusic

**Affiliations:** ^1^Center for Biological Sequence Analysis, Department of Systems Biology, Technical University of Denmark, 2800 Lyngby, Denmark; ^2^Department of Disease Systems Biology, Novo Nordisk Foundation Center for Protein Research, University of Copenhagen, 2200 Copenhagen, Denmark; ^3^Cancer Vaccine Center, Dana-Farber Cancer Institute, Harvard Medical School, Boston, MA 02115, USA; ^4^Bioinformatics Centre, Department of Biology, University of Copenhagen, 1017 Copenhagen, Denmark; ^5^Department of Computer Science, Metropolitan College, Boston University, Boston, MA 02215, USA; ^6^Department of Medicine, Harvard Medical School, Boston, MA 02115, USA; ^7^Laboratory of Immunobiology, Department of Medical Oncology, Dana-Farber Cancer Institute, Boston, MA 02115, USA

## Abstract

FluKB is a knowledge-based system focusing on data and analytical tools for influenza vaccine discovery. The main goal of FluKB is to provide access to curated influenza sequence and epitope data and enhance the analysis of influenza sequence diversity and the analysis of targets of immune responses. FluKB consists of more than 400,000 influenza protein sequences, known epitope data (357 verified T-cell epitopes, 685 HLA binders, and 16 naturally processed MHC ligands), and a collection of 28 influenza antibodies and their structurally defined B-cell epitopes. FluKB was built using a modular framework allowing the implementation of analytical workflows and includes standard search tools, such as keyword search and sequence similarity queries, as well as advanced tools for the analysis of sequence variability. The advanced analytical tools for vaccine discovery include visual mapping of T- and B-cell vaccine targets and assessment of neutralizing antibody coverage. FluKB supports the discovery of vaccine targets and the analysis of viral diversity and its implications for vaccine discovery as well as potential T-cell breadth and antibody cross neutralization involving multiple strains. FluKB is representation of a new generation of databases that integrates data, analytical tools, and analytical workflows that enable comprehensive analysis and automatic generation of analysis reports.

## 1. Introduction

An estimated 250,000–500,000 people die from seasonal influenza infection each year. The economic impact of influenza is immense due to the large number of lost working hours, hospitalizations, further medical complications, and treatment costs. Although vaccines against influenza exist, the rapid mutation of influenza virus calls for constant surveillance and annual vaccine reformulation [1]. A huge body of sequence data, annotations, and knowledge is available in the literature, online resources, and biological databases such as GenBank [2], UniProt [3], Protein Data Bank [4], EpiFlu Database [5], OpenFlu Database [6], Influenza Research Database (IRD) [7], and the Immune Epitope Database (IEDB) [8]. However, the underlying mechanisms of host/pathogen interaction are still not completely understood. The lack of a “universal” or broadly neutralizing influenza vaccine can be attributed to, among other factors, combinatorial complexity of the host immune system and the highly variable nature of viral antigens leading to immune escape of the emerging influenza variants [9, 10]. One approach, in an attempt to overcome challenges of immune escape, is to raise a T-cell response against class I or class II epitopes conserved among viral strains [11, 12]. Public databases represent valuable resource for the study and development of broadly protective T-cell vaccines, but our ability to analyze these data falls behind the pace of data accumulation.

Numerous computational analysis tools that are useful for vaccine target discovery are available. They include keyword and text search tools, sequence comparison tools such as the BLAST algorithm [13] or multiple sequence alignment tools such as MAFFT [14], MUSCLE [15], and the Clustal [16], 3D structure visualization tools [17, 18], HLA binding prediction algorithms [19–21], and conservation analysis tools [22, 23], among others. The application of these tools in discrete steps can yield valuable information; however the extraction of higher-level knowledge requires integrating data from multiple databases and employing various analytical tools to answer specific questions. For example, when a new infectious influenza strain emerges (such as H9N7 avian flu [24] or a new seasonal flu) it is desirable to rapidly investigate its similarities and dissimilarities with known sequences, its epidemic or pandemic potential in humans, how different it is from the past vaccine strains, and its T- and B-cell epitopes from previously circulating strains and estimate its immune escape potential. Additionally, for new pandemic strains (such as 2009 swine flu [25]) it is desirable to establish origin and identify strains that are useful vaccine candidates. Well-defined workflows enable rapid extraction of such knowledge and automated generation of reports that contain such information, for which knowledge-based systems have previously been utilized [26, 27]. The need for integration and advanced analysis of available data is rapidly increasing. The integration of multistep analysis of multidimensional data for vaccine analysis and discovery requires the automation of analytical workflows [28].

**Figure 1:**
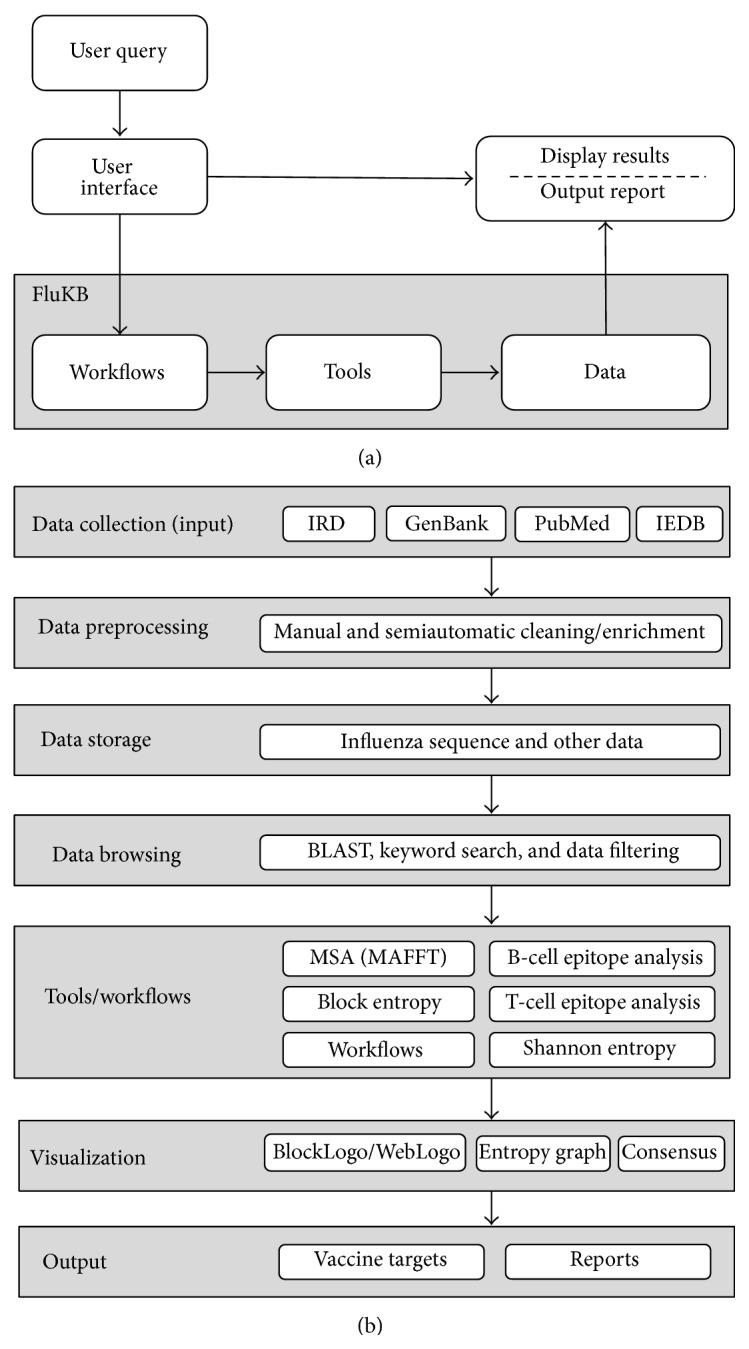
Overview of the architecture of FluKB. (a) Users can access FluKB through an interactive user interface where they can select specific data and tools or deploy a predefined workflow. (b) Top to bottom: data are collected, cleaned, and enriched. Higher-level knowledge extraction is enabled by utilization of tools assembled into workflows.

FluKB is a knowledge-based system that integrates multiple types of influenza data and analytical tools into such workflows to support vaccine target discovery. The datasets in FluKB consist of curated, enriched, and standardized protein sequence data, immunological data from multiple data sources, and a set of modular analysis tools. The analysis tools infrastructure comprises a library of individual tools along with standard (applicable to multiple pathogens) and specific influenza vaccine target discovery workflows. Furthermore, we developed a standardized nomenclature to enable and speed up data mining using automated workflows. FluKB has a user-friendly web-based interface to access the data, tools, predefined workflows, and workflow reports. The overall architecture of FluKB is shown in Figure 1.

## 2. Materials and Methods

### 2.1. KB-Builder

FluKB was implemented using the KB-builder framework [29]. Briefly, KB-builder consists of seven major functional modules that enable automated data extraction from multiple sources, data cleaning, import to a central repository, integration of basic analysis tools, integration of advanced analysis tools, workflow definition, and update and maintenance. The KB-builder framework enabled setting up a web-accessible knowledge base and the analysis workflows. A workflow takes the user request, performs complex analyses which combines specific data and analytical tools, and feeds the results into subsequent analyses to produce a comprehensive report. The web-accessible interface uses a set of graphical user interface forms. These interfaces access search routines, analytical tools, and workflows that use a combination of PHP, Perl, Common Gateway Interface (CGI), and C background software. Development of KB-build and FluKB was carried out in CentOS 5.11 Linux environment. The web server used is Apache HTTP server 2.2.3 so the access to the web server is per default parallelized for each user. The Linux server is a 16-core server with 32 GB ram and should be able to handle the traffic to the website.

### 2.2. Data Sources for FluKB Data Repository

The data repository within FluKB contains four types of data: protein sequence data, HLA-related data (T-cell epitopes and HLA ligands), 3D crystal structures, and neutralizing antibody-related data. Protein sequences available from Influenza A, B, and, C viruses were collected from IRD [7] and GenBank [2], while HLA-related data were collected from IEDB [8]. The complex structures of neutralizing antibodies against influenza virus hemagglutinin (HA) were collected from the Protein Databank (PDB) [4]. The binding and neutralization assays of each neutralizing antibody were collected from primary literature, as described in [30].

### 2.3. Data Collection, Cleaning, and Enrichment

#### 2.3.1. Protein Data Entries and Their Updates

**Figure 2:**
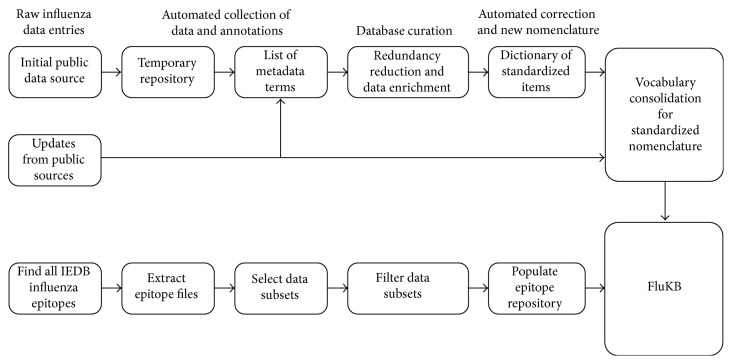
Semiautomated generation and updating of protein sequence repository of FluKB (upper path) and data extraction and repository creation of the influenza specific epitopes from IEDB (lower path).

The following sequence data and annotations were extracted from the IRD: protein sequence, GenBank identifiers (GI and Accession Numbers), UniProt ID, and nomenclature (type, host, location, ID, year, and subtype) when available. The metadata included a vocabulary that comprises the instances of the following terms: protein names, host names, geographic locations, years, and subtypes. The vocabulary was generated from the collected entries and enriched using primary literature. The vocabulary comprised correct terms as well as variants of the terms, and erroneous terms. During updates, each new entry is checked against the list of correct and erroneous terms. If all terms within the entry are matched to the correct terms, the entry is automatically annotated and converted into the FluKB format. If an erroneous term is identified, the curator is alerted and the correct term is proposed for the entry. The curator then approves the change or manually corrects the entry and updates the vocabulary, when needed. Each new term identified in the update dataset is inspected by the curator and added to the vocabulary. The vocabulary iteratively increases in size with each update and less than 20 new terms are usually identified for each update. This approach enables data curation that is of extremely high quality and can be performed very fast (Figure 2 upper path). We converted existing nomenclatures, whenever possible, into standardized formats. To verify the protein annotations of newly added entries, protein assignment of the strain proteomes was done by aligning the entry proteome sequence to a representative influenza reference sequences using the BLAST algorithm. We selected 10 proteins from UniProt that have detailed annotations as reference sequences for each protein of the two influenza types (A and B). The reference sequences are shown in Supplemental Table S1 (see Supplementary Material available online at http://dx.doi.org/10.1155/2015/380975).

#### 2.3.2. Immunological Data

The immunological data extracted from IEDB include T-cell epitope sequence, epitope type (HLA binding, naturally processed, or T-cell epitope), PubMed references, experimental methods and results, and HLA-allele restriction (Figure 2 lower path). FluKB includes entries from IEDB that bind HLA class I or II allele with at least one positive result, or those reported as T-cell epitopes.

#### 2.3.3. Neutralizing Antibody Data

For each neutralizing antibody, the following descriptive information is provided in the FluKB: isolation information (from human samples or from antibody phage-display libraries) from the primary literature, the corresponding crystal structure of antibody-HA complex from the PDB database, the B-cell epitope sequence variants detected from experimentally validated strains, and computationally defined B-cell epitopes on HA protein displayed as both sequence and 3D structure with Jmol [31].

### 2.4. Influenza Nomenclature Standardization and Definition of Data Mining Keys

Each influenza strain sample is annotated using the nomenclature originally proposed by the WHO [32] providing a shorthand description of influenza virus strains. However, the lack of a standardized vocabulary has made these nomenclatures error prone. For example, the nomenclature of an influenza isolate of type A, subtype H2N3, isolated from a duck in Heinersdorf in 1986, is written, using the original nomenclature as

 
*A/Peking duck/Heinersdorf/648-4/1986(H2N3)*

The lack of standardization of nomenclature has led to inconsistent nomenclature and incomplete metadata, thus increasing the difficulty in extraction of specific data subsets for analysis. The nomenclature in the Heinersdorf 1986 strain 648-4 has two issues. First the organism name term is erroneous (“Peking duck” is a traditional Chinese dish, while “Pekin duck” is the organism). Second, it is unclear where Heinersdorf is located. To ensure a complete access control over the sequence data within FluKB each entry was given a standardized data mining key. This key converts the nonstandardized nomenclature fields into a fully standardized format. The key is represented in FluKB as a standardized FASTA header that provides a condensed and detailed summary of the sample's information. The FluKB data mining key for the Heinersdorf sample HA protein is

 
*>FLU0175850|type:A|host:/Mallard;Anas platyrhynchos;8839|location:Heinersdorf;Berlin-Germany;DE-BE|isolate:648-4|year:1986|subtype:H2N3|protein:HA|seqtype:complete|variant:1|vaccine:_|genbank: CY117179|geneinfo: 386644010|uniprot:_|original: A/Peking duck/Heinersdorf/648-4/1986|key:yes*

This search key compresses detailed sequence annotations into the FASTA format enabling easy combination of the results of sequence comparison with the analysis of annotations. Standardized formats for host and geographic location enable proper grouping and mapping of results. For host species, the NCBI-Taxonomy IDs [33] and Bird Life International taxonomy (http://www.birdlife.org/datazone/info/taxonomy) names were used as standard terms, including the NCBI taxonomy number. For the geographical locations, two-letter ISO codes for the countries and provinces were used (ISO-3166, 2012) [33]. This allows for each of the host species and each geographic term to be described in nonambiguous terms. Examples of corrected ambiguities are shown in Table 1. The FASTA format is easily understandable because of the descriptive nature of the fields in the FASTA header. Finally we use the term* key:yes* for all entries that could be fully annotated allowing them to be utilized within the analysis framework of FluKB, while those that could not be fully annotated are assigned with* key:no* and can only be found by a search.

### 2.5. Implementation of Analytical Tools

A set of analytical and visualization tools have been integrated within the FluKB. These tools include a selection of keyword searching tools: MAFFT [14] for multiple sequence alignment (MSA) and BLAST [13] for sequence similarity search. Specialized tools for the analysis of variability include sequence conservation metrics and their visualization using block entropy analysis [34]. The T-cell epitope prediction tools for HLA Class I and Class II have been integrated within FluKB for vaccine-related analyses. WebLogo [22] and BlockLogo [23] tools are used for visualization of results.

#### 2.5.1. Sequence Conservation Metrics

FluKB enables conservation analysis of single positions within protein sequences, of linear blocks of amino acids extracted from multiple sequence alignments (MSA) of proteins using block entropy [34]. In addition, virtual peptides can be constructed from discontinuous epitopes within MSA and can be analyzed using block entropy, enabling the variability analysis of B-cell epitopes [30]. All these calculations are based on the calculation of Shannon entropy [35].

#### 2.5.2. Visualization of Sequence Variability

WebLogo enables fast and easy interpretation of the position specific variability in an alignment. It displays amino acids by their corresponding one-letter code on a graph where all the amino acids present in one position are stacked on top of each other, and the frequency in the position is then based on their individual height in the graph. BlockLogo enables variability analysis of peptides (either linear peptides or discontinuous strings of amino acids), rather than single residues. The combinatorial number of possible blocks that can be created from a WebLogo can quickly become very large because of variation in individual positions. BlockLogo only shows the exact peptides that are most frequent for the investigated positions.

#### 2.5.3. Prediction of MHC Class I and II Binders

For class I HLA binding prediction, the NetMHCpan 2.8a algorithm [19] was implemented. The alleles available for predictions are HLA-A∗02:01, -A∗03:01, -A∗11:01, -A∗2402, -B∗07:02, -B∗08:01, and -B∗15:01, since prediction accuracies for these alleles are relatively high [36]. Additionally, we predict binders to HLA-A∗01:01 since this allele is of very high frequency in the human population, although these predictions are of slightly lower accuracy than the seven benchmarked alleles. Collectively these alleles cover approximately 82% of human population [37]. For class II MHC predictions we used NetMHCIIpan 3.0 [21], with which users can predict binders to HLA-DRB1∗01:01, ∗03:01, ∗04:01, ∗07:01, ∗11:01, ∗13:01, and ∗15:01, as these have been benchmarked and validated for high accuracy [21]. These alleles cover approximately 40% of the human population [37].

#### 2.5.4. Analysis of Sequence Similarity and Geographical Mapping (Strain Mapper)

The standardized data mining key in FluKB enables sequence similarity searching and the display of a sequence's origin on the world map. For this purpose, we developed the specialized tool, Strain Mapper. The query sequence is entered in the search window and optionally the maximal number of amino acid mismatches can be selected. We used Google Earth software (http://www.google.com/earth/) for geographic map display. It is combined with sequence similarity analysis to allow user to select a query sequence and find its closest matches in FluKB and visualize them on the world map. This feature provides visualization of epidemiological information useful for evaluating the spread of a given virus and closely related variants.

## 3. FluKB Database

### 3.1. Data Repository

The FluKB sequence repository as of December 2014 contains 402,306 sequence entries from 75,426 unique strains of influenza. There are 67,907 type A, 7,028 type B, 194 type C, and 297 unknown type sequence entries. Out of 330,435 full-length sequences and 71,501 fragments, 370 protein sequences failed to align well to any of the template alignments during the annotation process. Each sequence entry contains information about location, host, and time of isolation, as well as a standardized nomenclature for identification of strains. Each entry contains a protein sequence with standardized, curated, and enriched annotations (Table 2).

The epitope repository contains a total of 357 unique T-cell epitopes (194 class I and 163 class II) and 685 unique HLA binders (572 class I and 113 class II). Each record describes the type of epitope (T-cell epitope, naturally processed, or HLA binder), epitope sequence type (only exact epitopes are included in the repository), experimental method used for validation, HLA-restriction, and literature references.

Twenty-eight neutralizing antibodies against influenza virus have crystal structures of HA/antibody complexes available in PDB. The functional data and neutralizing specificity of these antibodies were collected from published articles. Twenty of these antibodies target the globular head of the HA protein, and the binding sites of the remaining eight antibodies are located on HA stem region. All of these antibodies were classified as broadly neutralizing (cross neutralization within subtype or across subtypes) and strain-specific antibodies.

We plan to have yearly updates of FluKB moving forward as new data and tools become available.

### 3.2. Data Cleaning, Quality Control, and Enrichment

The sequence data collected from IRD were subject to extensive cleaning, quality control (QC), and enrichment of annotations. We found that 142,232 (38.25%) of the 402.306 entries contained at least one type of error, ambiguity, or missing data. Most errors were in the geographic location fields where 72,340 (17.9%) had an error and 6,821 (12.1%) had missing information in the entry (see Table 3). In the initial screen of the data we found 2,977 entries that did not conform to nomenclature standard, including 305 entries that lacked information about host species, 867 entries that lacked separation fields within the nomenclature, and 1,805 other deficiencies. These entries were manually corrected and their nomenclatures were updated.

Furthermore, abbreviations, alternative, and misspelled names constituted the largest proportion of errors and were present in more than 10,000 entries. All name-related errors were corrected by the dictionary consolidation using the dictionary of standardized metadata terms. An example of the redundancy is shown in Table 1 where the host “Mallard” is found in 16,457 of the FluKB entries described by 14 different terms. In total, 96.41% of errors of various types described above were corrected and 469 standardized forms of missing data (such as location and host species) were added by manually searching the original literature.

Our effort in the data cleaning and enrichment stage focused on minimizing errors and maximizing data completeness to enhance knowledge extraction for discovery of potential vaccine targets in influenza, as well as genetic and epidemiological modeling of viral strains. Because of the system of reference sequences, templates, and reference MSA implemented in FluKB, we expect that the majority of future entries will be automatically corrected, if they contain errors and redundancies already encountered by the system. Any new errors will be subject to manual curating and updating of dictionaries.

### 3.3. Standardized Nomenclature

To enable automated data mining and workflows, we created data mining keys from the original nomenclature of influenza viruses with standardized terms. The data mining keys utilized NCBIs taxID database for host species [32] and the ISO codes for geographical location (ISO-3166, 2012) [33]. A total of 398,078 sequences (98.95%) were assigned the new developed nomenclature, while 4,228 (1.05%) could not be assigned. The original standard nomenclature is included in the data mining key as a reference for additional literature searches and text mining of article databases [37]. Data mining for vaccine targets often requires the analysis of subsets of data, for example, patient data such as specific HLA profile, age group, phenotypes, or other factors. Similarly, epidemiological modelling may need analysis of sequences from certain hosts, for example, specific migrating birds, or limited to geographical locations. The host, time, and location of collection are key information that help determine the spread of specific influenza strains and are central for better understanding of influenza outbreaks [38]. The data mining keys enable such analyses by having a standardized nomenclature, which pattern recognition algorithms can utilize as labels. Entries without the data mining key are made unavailable to the analysis on FluKB as inclusion of entries that lack data could affect the reliability and the outcome of the results. The data mining keys are furthermore nomenclature crucial for the automation of computational analyses; standardization of nomenclature fields allows the computer to interpret the data automatically, which previously was limited. For instance, the taxonomy ID of hosts enables host specific analyses that can potentially reveal features important for interspecies transmission of influenza. Proper organization of data allows for grouping of data by ancestral species and the variability can be followed over time. Furthermore the ISO codes for the geographical location by country and provinces enhance analyses in, for instance, epidemiological studies where an increased resolution in terms of actual spread can be analysed. This information can be used for the analysis of changes in T-cell and B-cell epitopes.

## 4. FluKB Tools

### 4.1. Database Searching and Querying

In FluKB, two search strategies can be deployed for sequence search: annotation-based or epitope-based. The first is a keyword search that enables the user to extract the data of FluKB into specific subsets and the second is a sequence similarity search by BLAST [13]. These search types are vital for the following data analysis as they enable the user to select the needed datasets based on specific scientific questions.

#### 4.1.1. Keyword Search

**Figure 3:**
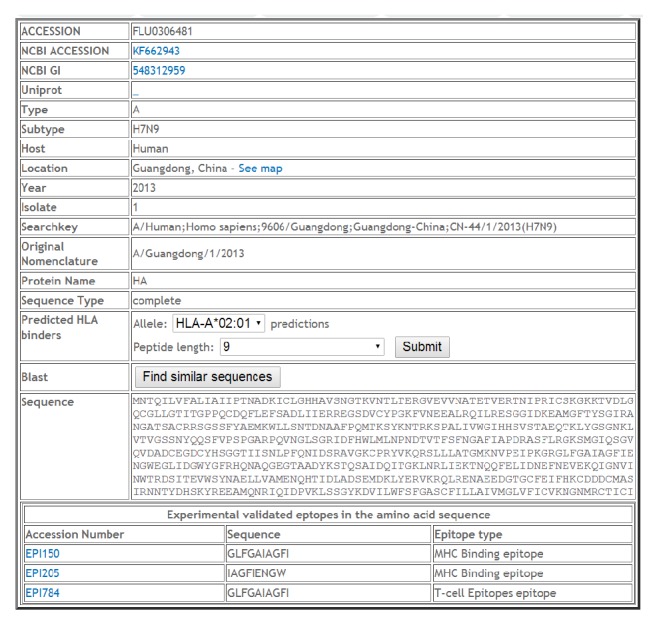
Record FLU0306481, Protein HA representing the 2013 H7N9 outbreak of bird flu in China.

The user can query the sequence entries for information such as the type, protein, subtype, year range, country, province, host, original nomenclature, and sequence type (fragment or full protein) by keyword search. The sequence entry database is indexed in order to decrease the retrieval time. An example of entry page retrieved by ID FLU0306481 or Strain Name A/Guangdong/1/2013, protein HA, is shown in Figure 3.

#### 4.1.2. Sequence Similarity Search

FluKB has an indexed database generated from sequence entries that can be searched for sequence similarity using BLAST algorithm. The standard parameters are used: *E* value (10), word size (≥2), substitution matrix (BLOSOM62), gap cost (11) and extension (1), size of the result list (500), and pairwise list (250). Besides sequence search, the FluKB entries can be searched for T-cell epitopes and B-cell epitopes.

#### 4.1.3. Stand-Alone Tools

FluKB can be queried using a selection of analytical tools under the tab “Tools.” Sequence alignment by MSA can be performed under the “Sequence alignment” tab by entering either a list of sequence IDs in the query window or a selection of subsets of proteins by name, influenza type, subtype, range of years of identification, country, province, host, or complete sequences/fragments. The protein subsets selection window (Supplemental Figure S1) can be used for sequence variability analysis and block entropy calculation under appropriate tabs. Epitope block entropy (T-cell epitopes) uses protein subsets selection window that enables the input of epitope sequence. The strain mapping tool is described in Section 2.5.4.

### 4.2. Variability Analysis

The analysis of variability of viral sequences is important for understanding the emergence of new strains, immune escape, changes in pathogenicity, the extent of spread of viral strains, and vaccine design. The variability analysis can be performed interactively, but the variability analysis tools are also integrated in the T-cell and B-cell mapping tools and relevant workflows. The interactive variability analysis can be performed using individual sequences or sets of sequences. The tools used in variability analysis are shown in Table 4. The main tools for the analysis of variability are BLAST search that can be accessed from individual entries (Figure 3, “find similar sequences”) or from the “Sequence alignment” under the “Tools” tab. The MSA can be performed from the results of BLAST search by selection of sequences and clicking the “Align them…” key. The positions of variability within the MSA results are color-coded for better visual inspection and each sequence is hyperlinked to its record (Supplemental Figure S2). The “Sequence variability analysis” tool plots entropy (red curve) and the percentage of sequences (blue curve) containing the consensus amino acid at all positions along with the consensus sequence (Supplemental Figure S3). Further visualization can be achieved by “Block entropy” calculation, which visualizes the conservation of peptides of lengths appropriate for immune recognition, rather than individual residues [34]. The “Epitope block entropy” calculation displays variability of a specific epitope across the selected subset of sequences.

### 4.3. T-Cell Epitope Search

**Figure 4:**
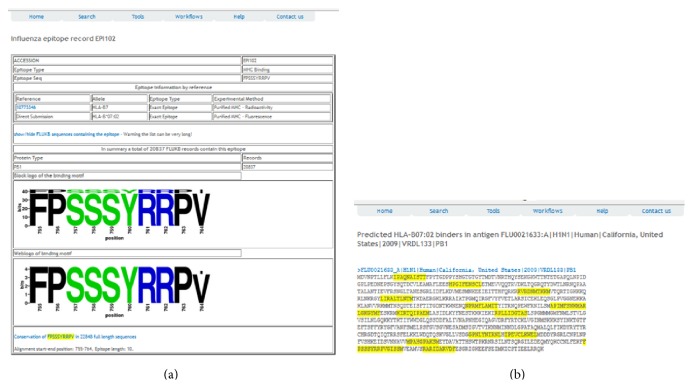
T-cell epitope EPI102 and the T-cell epitope analysis from the record FLU0021633. (a) Entry EPI150 with the graphical display of present T-cell epitope variants. (b) Graphical display of predicted T-cell epitopes (HLA-B∗07:02, length 10, IC_50_ < 1000 nM).

T-cell epitope analysis can be performed directly from the protein entry. For example, three T-cell epitope entries are displayed on the record entry page (Figure 3). The epitope entry page for EPI150 is shown in Figure 4(a). In addition, the prediction of T-cell epitopes can be performed by selecting allele and peptide length in the “Predicted HLA Binders” field followed by the “Submit” action. The visual display of experimentally verified T-cell epitopes is shown in Figure 4(b).

T-cell epitope search can also be initiated from the “Search” tab, where epitope can be searched by the sequence. The results will appear as a list of epitopes along with their binding or T-cell restriction specificities. The list is hyperlinked to the epitope record, an example of which is shown in Figure 4(a). Finally, T-cell epitope search can be performed using the workflow titled “Vaccine targets” under the Workflows tab. After selection of input parameters, for example, Allele “HLA-A∗0201,” Protein “HA,” Influenza type “A,” subtype “H7 N9,” year(s) “2013 2014,” Affinity threshold “500 nM,” and Conservation Threshold “95%” (and remaining values “default”), 98 sequences will be selected and the report will be generated.

### 4.4. B-Cell Epitope Search

B-cell epitope analysis can be performed from the Search tab by selecting “Antibodies list.” By the end of May, 2014, 28 antibodies and their detailed neutralizing and structural information have been deposited in FluKB. All of these antibodies are neutralizing antibodies against hemagglutinin protein on influenza virus. These antibodies are listed on the webpage, while their respective B-cell epitopes can be displayed on three interactive structures: X-31 strain-specific antibodies on HA structure from 1KEN, broadly neutralizing antibodies on HA structure from 1EO8, and influenza B virus antibodies on HA structure from 4FQK. This feature enables visual comparison of antibody-specific B-cell epitopes.

**Figure 5:**
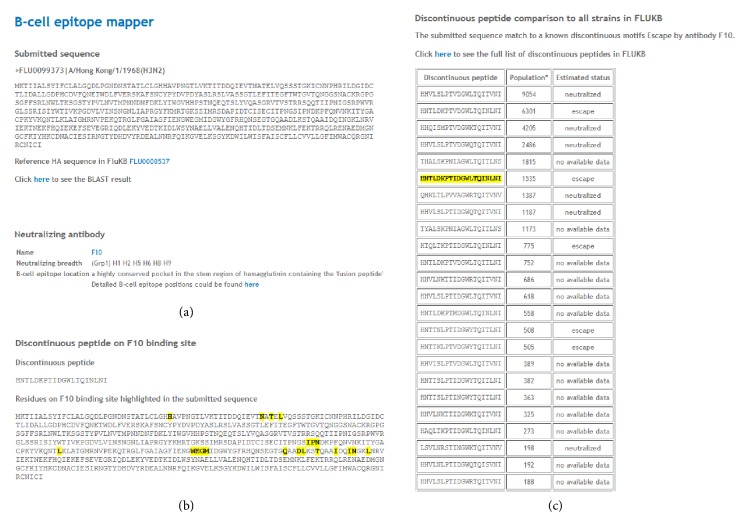
The results of B-cell epitope analysis II. Analysis of entry FLU0099373 for broadly neutralizing antibody F10 interaction. (a) The BLAST result of the query sequence to the sequence with highest identity in FLUKB, (b) the discontinuous peptide extracted from the query sequence and respective residue positions highlighted in full sequence, and (c) the summary information of neutralizing antibody. The discontinuous peptide from the query sequence is compared to all discontinuous peptides generated from FLUKB (with their neutralizing status listed). The identical sequence is highlighted in yellow with estimated status of neutralization given.

For each neutralizing antibody, the isolation information, structure information, and computational identified B-cell epitope information can be accessed. Also, the neutralized motifs and escape motifs extracted from experimentally validated strains from the primary literature are presented as well [30]. In addition, two workflows have been implemented for further analysis: the neutralization coverage estimation and B-cell epitope mapping (Supplemental Figure S4). The neutralized/escape coverage by a specific existing neutralizing antibody is calculated for the complete population of influenza strains. The strain population coverage by a neutralizing antibody can be assessed within any selected subset of influenza strains, such as year range, specific subtype, and geographic coverage. The B-cell epitope mapping is performed by submitting a query hemagglutinin sequence. Cross neutralization coverage of a known neutralizing antibody can be estimated based on sequence comparison to the known neutralizing epitopes. A discontinuous peptide is extracted based on epitope positions determined from crystal structures. This tentative discontinuous peptide is then compared to the B-cell epitopes of experimentally validated strains. An example of B-cell epitope analysis is shown in Figure 5.

FluKB offers the capability to address complex questions relating to sequence variability on very specific subsets, identification of potential T-cell epitopes, and selection and combination of these epitopes into polyvalent vaccine constructs. The modular structure of the workflow renders FluKB highly flexible. The tools and data can be reorganized and more tools can be created to answer additional questions, for example, relating to epidemiological modeling and analysis of cross protective potential of neutralizing antibodies. The overall architecture can be viewed in Supplemental Figure S5.

## 5. Conclusion

Publically available influenza data are a valuable resource for computational analyses with applications in vaccine design. Similarly, existing bioinformatics tools provide the means for extraction of information and new knowledge. However, to utilize the full potential of these resources, data preprocessing must be performed and analytical tools must be carefully combined into well-defined workflows. These workflows allow users to ask specific questions (scientific, technical, and clinical) and provide means for systematic data analysis. These workflows can automatically generate comprehensive analysis reports. The infrastructure of data and tools is the backbone of FluKB and similar knowledge-based systems [26, 27].

Despite many years of research and available vaccines, influenza remains a major public health burden and a threat of a major new pandemic. Multiple data sources provide information on protein and nucleotide sequences and immune epitopes in influenza [2, 5–8, 39, 40]. They represent well-maintained catalogues of influenza sequences and annotations, along with a selection of basic search tools. They focus mainly on providing access to data, extraction, and simple analyses. The FluKB was developed focusing on a different purpose, the facilitation of data mining for influenza vaccinology and immunology of influenza infection. The FluKB has very clean and standardized data, integrating information on antigen sequences, and immunological epitopes. The set of integrated analysis tools and workflows are designed to aid rational vaccine design. This includes the discovery of vaccine targets, assessment of variability, and in-depth analysis of immune epitope. FluKB is a unique data mining system for largely automated knowledge discovery from the ever-increasing body of influenza data with applications in both T-cell and B-cell immunology and vaccinology.

Systematic discovery of influenza vaccine targets requires highly accurate, up-to-date, and standardized data of influenza antigens and immune epitopes. The sequence and epitope data available through publications, various reports, and databases vary in quality, granularity, and data formats. The extraction of knowledge and discovery of vaccine targets from diverse and scattered data sources are a challenging and time-consuming task. FluKB integrates the content and the analytical tools in a unified system that enables the automation of complex queries and discovery. FluKB is a contribution to the long-standing quest for universal influenza vaccines [41, 42] by allowing a large-scale analysis on a large collection of annotated influenza sequences. FluKB is publicly available at http://research4.dfci.harvard.edu/cvc/flukb/.

## Supplementary Material

The supplementary material displays screenshots of the sequence variability tool (S1), the colored display of MSA in the browser (S2), and the consensus and Shannon Entropy graph of a MSA (S3). The supplementary material also describes the collection process of the neutralizing antibodies and the two workflows “The neutralization coverage estimation” and “B-Cell Epitope Mapper” (S4). Finally, the supplementary materials contains the reference sequences used for the protein annotation in FluKB (S5).

## Figures and Tables

**Table 1:** All the alternative instances of the host Mallard in all of the entries in the knowledge base of FluKB. The standardized name for the search key is *Mallard*; *Anas platyrhynchos*; *8839. *

Alternative names for Mallard	Number of times present in dataset	Status

*Anas domesticus *	1	Ambiguous
*Anasplatyrhynchos *	9	Error
*Anasplatyrhynchos *	39	Correct
Domestic duck	58	Variant
Domestic Mallard	11	Variant
Feral duck	1	Ambiguous
Khaki campbell duck	12	Variant
Mallard	25,844	Correct
Mallard duck	1,172	Redundant
Pekin duck	137	Variant
Peking duck	92	Error
Sentinel duck	13	Error
Wdk	11	Ambiguous (abbreviation)
Wild duck	952	Ambiguous

Total	28,352	

**Table 2:** The data fields in each protein entry.

Field title	Field content
CVC accession	Accession number unique to FluKB
NCBI accession	Accession number unique to NCBI
GenBank GI	Accession number unique to GenBank
Type	The influenza virus type
Subtype	The serotype
Host	Host of collection
Country	Location of collection
Year	Time of collection
Isolate	Isolate name
Vaccine strain	Years the strain has been used as a vaccine strain
Original nomenclature	The original nomenclature from the raw data
Protein name	The protein name, based on template BLAST
Sequence type	Full or fragment of the protein sequence
Predict HLA binders	Predict HLA binders to the sequence
Blast sequence	Blast the sequence for similar sequences of FluKB
Sequence	The amino acid sequence of the entry
Epitopes in sequence	IEDB epitopes found in the protein sequence

**Table 3:** The types of errors found for the geographical location in the metadata of the entry.

Type of error	Number
Case errors	26,335
Redundant information	14,165
Alternative name and abbreviations	11,208
Misspellings	2027
Alternative spellings	9,112
True location could not be determined	9493

Total	72,340

**Table 4:** The sources of the integrated tools in FluKB and URL for their stand-alone versions web services.

Tool	URL	Reference
BLAST	http://blast.ncbi.nlm.nih.gov/Blast.cgi	[11]
MAFFT MSA	http://mafft.cbrc.jp/alignment/software/	[12]
NetMHCpan 2.8a	http://www.cbs.dtu.dk/services/NetMHCpan/	[15]
NetMHCIIpan 3.0	http://www.cbs.dtu.dk/services/NetMHCIIpan/	[19]
Block entropy	http://research4.dfci.harvard.edu/cvc/flukb/HTML/blockentropy.php	ISO-3166
BlockLogo	http://mafft.cbrc.jp/alignment/software/	[21]

## References

[1] (2013). Prevention and control of seasonal influenza with vaccines: recommendations of the advisory committee on immunization practices—United States, 2013-2014.. *Morbidity and Mortality Weekly Report*.

[2] Benson D. A., Karsch-Mizrachi I., Lipman D. J., Ostell J., Sayers E. W. (2009). GenBank. *Nucleic Acids Research*.

[3] The UniProt Consortium (2012). Reorganizing the protein space at the Universal Protein Resource (UniProt). *Nucleic Acids Research*.

[4] Berman H. M., Westbrook J., Feng Z. (2000). The protein data bank. *Nucleic Acids Research*.

[6] Liechti R., Gleizes A., Kuznetsov D. (2010). OpenFluDB, a database for human and animal influenza virus. *Database*.

[7] Squires R. B., Noronha J., Hunt V. (2012). Influenza research database: an integrated bioinformatics resource for influenza research and surveillance. *Influenza and other Respiratory Viruses*.

[8] Vita R., Zarebski L., Greenbaum J. A. (2010). The immune epitope database 2.0. *Nucleic Acids Research*.

[9] Heiny A. T., Miotto O., Srinivasan K. N. (2007). Evolutionarily conserved protein sequences of influenza a viruses, avian and human, as vaccine targets. *PLoS ONE*.

[10] Sitaras I., Kalthoff D., Beer M., Peeters B., de Jong M. C. M. (2014). Immune escape mutants of highly pathogenic avian influenza H5N1 selected using polyclonal sera: identification of key amino acids in the HA protein. *PLoS ONE*.

[11] Furuya Y., Chan J., Regner M. (2010). Cytotoxic T cells are the predominant players providing cross-protective immunity induced by *γ*-irradiated influenza A viruses. *Journal of Virology*.

[12] Keskin D. B., Reinhold B. B., Zhang G. L., Ivanov A. R., Karger B. L., Reinherz E. L. (2015). Physical detection of influenza A epitopes identifies a stealth subset on human lung epithelium evading natural CD8 immunity. *Proceedings of the National Academy of Sciences*.

[13] Altschul S. F., Gish W., Miller W., Myers E. W., Lipman D. J. (1990). Basic local alignment search tool. *Journal of Molecular Biology*.

[14] Katoh K., Misawa K., Kuma K.-I., Miyata T. (2002). MAFFT: a novel method for rapid multiple sequence alignment based on fast Fourier transform. *Nucleic Acids Research*.

[15] Edgar R. C. (2004). MUSCLE: multiple sequence alignment with high accuracy and high throughput. *Nucleic Acids Research*.

[16] Larkin M. A., Blackshields G., Brown N. P. (2007). Clustal W and Clustal X version 2.0. *Bioinformatics*.

[19] Lundegaard C., Lamberth K., Harndahl M., Buus S., Lund O., Nielsen M. (2008). NetMHC-3.0: accurate web accessible predictions of human, mouse and monkey MHC class I affinities for peptides of length 8-11. *Nucleic Acids Research*.

[20] Nielsen M., Justesen S., Lund O., Lundegaard C., Buus S. (2010). NetMHCIIpan-2.0—improved pan-specific HLA-DR predictions using a novel concurrent alignment and weight optimization training procedure. *Immunome Research*.

[21] Nielsen M., Lund O. (2009). NN-align. An artificial neural network-based alignment algorithm for MHC class II peptide binding prediction. *BMC Bioinformatics*.

[22] Crooks G. E., Hon G., Chandonia J.-M., Brenner S. E. (2004). WebLogo: a sequence logo generator. *Genome Research*.

[23] Olsen L. R., Kudahl U. J., Simon C. (2013). BlockLogo: visualization of peptide and sequence motif conservation. *Journal of Immunological Methods*.

[24] Kageyama T., Fujisaki S., Takashita E. (2013). Genetic analysis of novel avian A(H7N9) influenza viruses isolated from patients in China, February to April 2013. *Eurosurveillance*.

[25] Peiris J. S. M., Poon L. L. M., Guan Y. (2009). Emergence of a novel swine-origin influenza A virus (S-OIV) H1N1 virus in humans. *Journal of Clinical Virology*.

[26] Olsen L. R., Zhang G. L., Reinherz E. L., Brusic V. (2011). FLAVIdB: a data mining system for knowledge discovery in flaviviruses with direct applications in immunology and vaccinology. *Immunome Research*.

[27] Zhang G. L., Riemer A. B., Keskin D. B., Chitkushev L., Reinherz E. L., Brusic V. (2014). HPVdb: a data mining system for knowledge discovery in human papillomavirus with applications in T cell immunology and vaccinology. *Database*.

[28] Söllner J., Heinzel A., Summer G. (2010). Concept and application of a computational vaccinology workflow. *Immunome Research*.

[29] Zhang G. L., Chitkushev L., Olsen L. R., Kudahl U. J., Simon C., Brusic V., Sakharkar M. (2014). Streamlining the development of immunological knowledge bases. *Genomics Drug Discovery*.

[30] Sun J., Kudahl U. J., Simon C., Cao Z., Reinherz E. L., Brusic V. (2014). Large-scale analysis of B-cell epitopes on influenza virus hemagglutinin—implications for cross-reactivity of neutralizing antibodies. *Frontiers in Immunology*.

[31] Hanson R. M. (2010). Jmol-a paradigm shift in crystallographic visualization. *Journal of Applied Crystallography*.

[32] WHO (1980). A revision of the system of nomenclature for influenza viruses: a WHO memorandum. *Bulletin of the World Health Organization*.

[33] Federhen S. (2012). The NCBI Taxonomy database. *Nucleic Acids Research*.

[34] Olsen L. R., Zhang G. L., Keskin D. B., Reinherz E. L., Brusic V. (2011). Conservation analysis of dengue virust-cell epitope-based vaccine candidates using peptide block entropy. *Frontiers in Immunology*.

[35] Shannon C. E. (1948). A mathematical theory of communication. *The Bell System Technical Journal*.

[36] Lin H. H., Ray S., Tongchusak S., Reinherz E. L., Brusic V. (2008). Evaluation of MHC class I peptide binding prediction servers: applications for vaccine research. *BMC Immunology*.

[37] Gragert L., Madbouly A., Freeman J., Maiers M. (2013). Six-locus high resolution HLA haplotype frequencies derived from mixed-resolution DNA typing for the entire US donor registry. *Human Immunology*.

[38] Onozuka D., Hagihara A. (2008). Spatial and temporal dynamics of influenza outbreaks. *Epidemiology*.

[39] Consortium U. (2010). Ongoing and future developments at the Universal Protein Resource. *Nucleic Acids Research*.

[40] Rose P. W., Bi C., Bluhm W. F. (2013). The RCSB Protein Data Bank: new resources for research and education. *Nucleic Acids Research*.

[41] McMichael A. J., Gotch F. M., Noble G. R., Beare P. A. S. (1983). Cytotoxic T-cell immunity to influenza. *The New England Journal of Medicine*.

[42] Sui J., Hwang W. C., Perez S. (2009). Structural and functional bases for broad-spectrum neutralization of avian and human influenza A viruses. *Nature Structural and Molecular Biology*.

