# Supplementary material for: FluKB: A Knowledge-Based System for Influenza Vaccine Target Discovery and Analysis of the Immunological Properties of Influenza Viruses
Source: J Immunol Res. 2015 Oct 4;2015:380975. doi: 10.1155/2015/380975 (PMC4609449; doi:10.1155/2015/380975)
Supplement: Supplementary file 1 — The supplementary material displays screenshots of the sequence variability tool (S1), the colored display of MSA in the browser (S2), and the consensus and Shannon Entropy graph of a MSA (S3). The supplementary material also describes the collection process of the neutralizing antibodies and the two workflows “The neutralization coverage estimation” and “B-Cell Epitope Mapper” (S4). Finally, the supplementary materials contains the reference sequences used for the protein annotation in FluKB (S5). [file 380975.f1.pdf]

Supplemental Figures

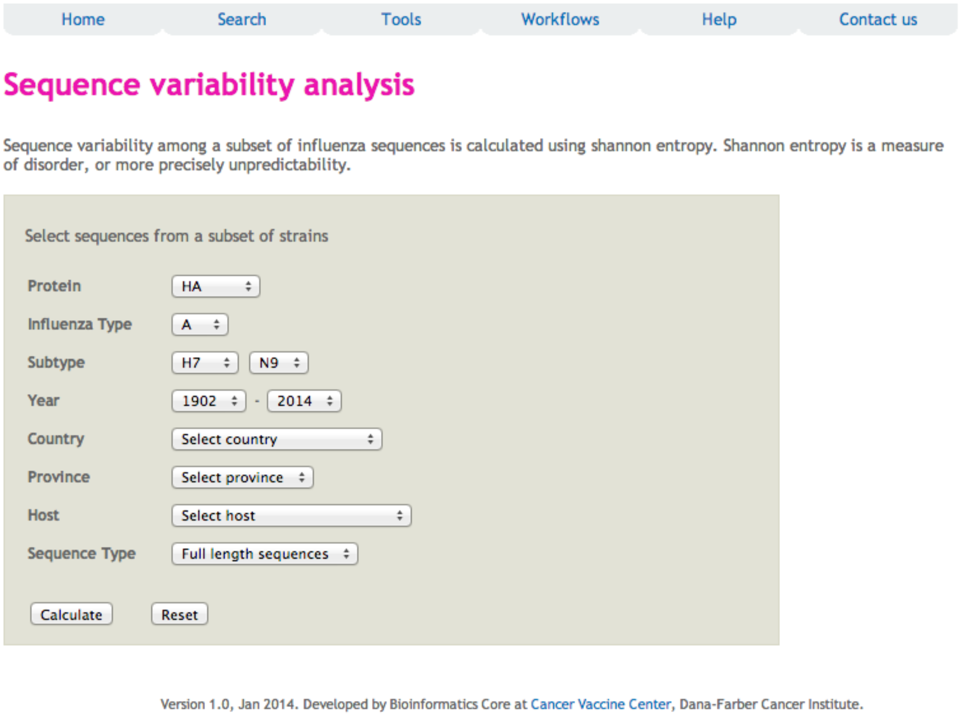

Supplemental Figure S1: The protein selection window that can be used to subset and filter the protein data in any way the user may prefer.

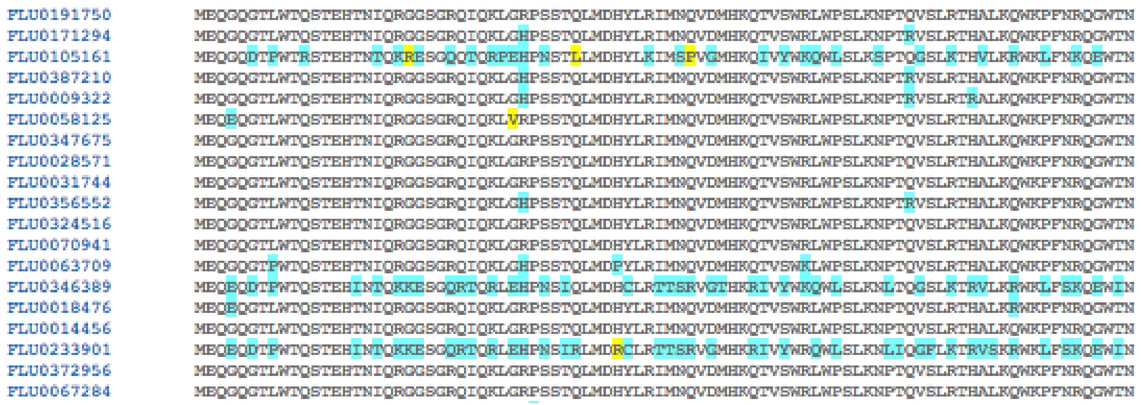

Supplemental Figure S2: The result of a query using the protein selection window that can be used to select a specific subset of protein data using various combinations of protein, influenza type, subtype, range of years of identification, geographic origin (country, province), host, and sequence type.

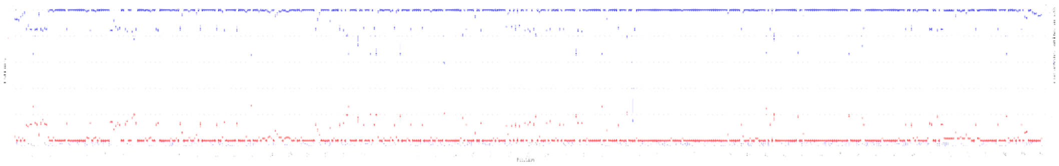

Supplemental Figure S3: An example output from the sequence variability analysis. The blue line display the percentage of sequences containing the consensus amino acids at a particular position in the alignment. The red line is the entropy of a given position in the alignment calculated by Shannon entropy. The example is a run from the setting shown in Supplemental Figure S1.

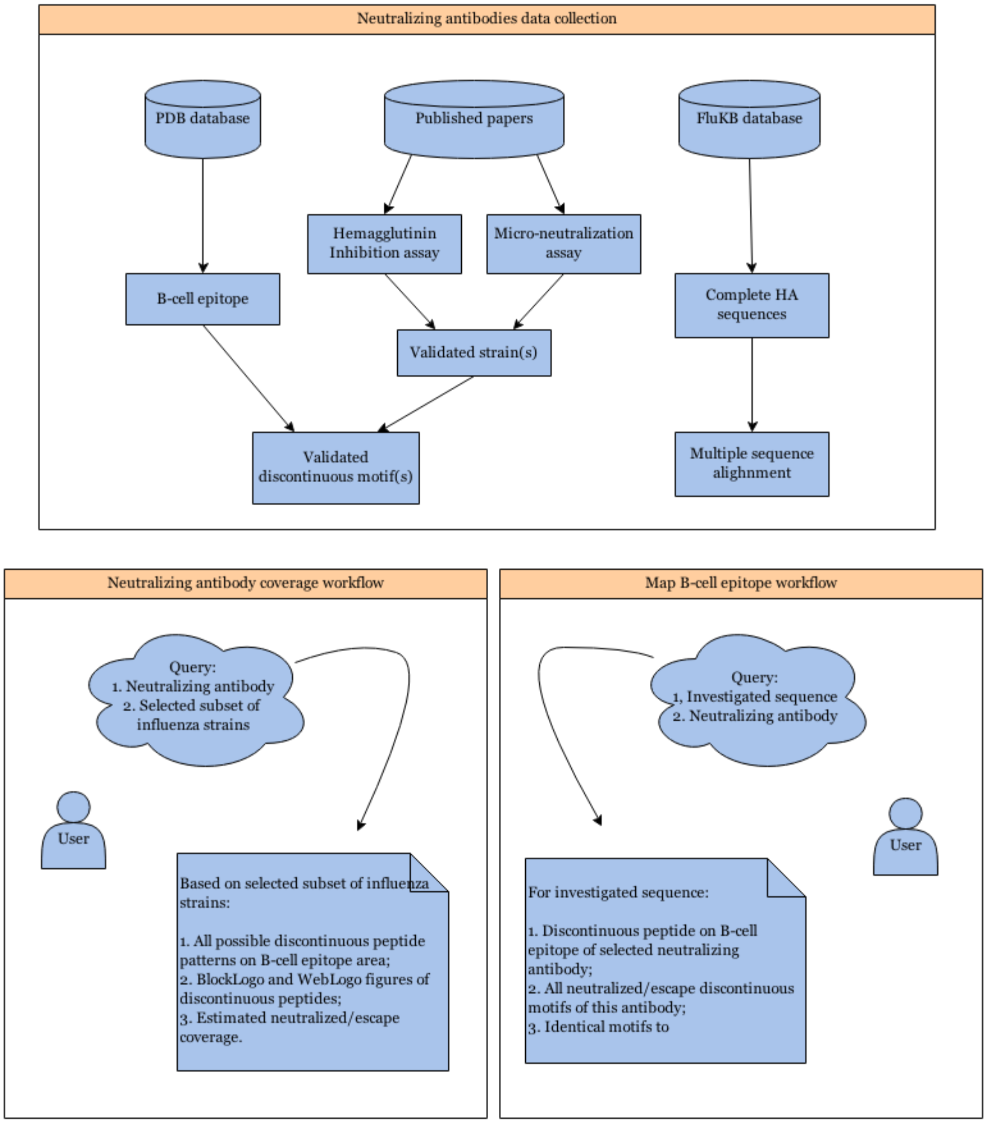

Supplemental Figure S4: Neutralizing antibodies-related data collection and two antibody analysis workflows. The upper panel shows the process of collection of neutralizing antibody-related information.

Supplemental Tables

Supplemantal Table S1: The list of GenBank accession numbers for the influenza reference sequences used in FluKB for protein annotations.

**GB accession number for Influenza Virus A**	
**HA**	M55059 AB269693 DQ508897 M25290 X05907 M16740 AF091310 AY383755 AB275283 CY015073
**M1**	M63530 AY210059 M63522 CY015109 AF144306 CY009205 CY034125 CY021054 CY019796 CY006708
**M2**	CY014695 X08093 CY020438 CY034125 M63530 M65019 M63521 CY015104 M63529 CY006836
**NA**	K01393 AB276110 AB124654 D00715 D00715 AF250363 M38335 CY009206 CY020463 CY019797
**NP**	M27298 M27520 L07352 L07351 D00050 M22573 M30748 CY009207 CY005674 CY034127
**NS1**	CY016240 CY026215 CY034128 M25372 M25373 AY651565 AY651568 M80973 K00578 AF348198
**NS2**	AB275287 AF001663 CY020297 CY034128 CY009208 CY006711 CY026295 CY002500 M25372 M25369
**PA**	M26078 CY014699 M26084 NC_007376 AY210006 CY005676 CY009353 CY008681 CY026216 CY034129
**PA-X**	P0DJW9 P0CK84 P0CK92 P0CK71 P0DJU2 P0DJW6 P0DJV5 P0DJS7 P0DJV3 P0CK87
**PB1**	M25929 M25930 M25931 M38376 M25935 DQ321291 AY651676 CY015113 CY015107 CY019801
**PB1-F2**	CY034130 CY009210 CY020443 CY014677 CY015095 CY014777 CY003742 CY007625 CY006050 CY002502
**PB2**	M38277 M73517 M73521 M73513 AY059525 AF348170 AY651751 CY034131 CY026218 CY009211
**GB accession number for Influenza Virus B**	
**NS2**	AB120446 AY582078 AY582083 AB120449 AF100399 AY687395 AY582075 AY582080 AY582081
**BM2**	M20175 M20176 AY260941 M14909 JN899476 AY581983 EU305617 JN899470 AY581975 AJ783374
**HA**	K00425 K00423 X17222 K00424 X53098 K02713 M58428 X00897 X13551 X13553
**M1**	AY260941 NC_002210 M20175 M20176 M14909 AF077348 AY581975 AY581978 AY581978 AY581980
**NA**	M54967 J02095 M30632 M30633 M30634 M30635 M30636 M30637 M30638 M30639
**NB**	J02095 M30637 M30633 M54967 M30631 M30632 M30634 M30635 M30636 M30639
**NP**	NC_002208 K01395 X14217 M20173 M20174 AF005739 K01139 AF100367 AB036876 AY582020
**NS1**	M19796 M19788 M19790 M19792 M20225 M20224 M19786 M19795 M19794 M19791
**PA**	NC_002206 M16711 M20171 M20172 AF005738 CY018738 CY018530 CY038292 EF626641 AY582032
**PB1**	M20479 NC_002204 M20169 AF005736 CY115117 CY119656 CY119560 CY119864 CY119832 CY119928
**PB2**	AF101982 M20163 M20168 AF005737 AY582060 AY582066 EU305612 EF626643 CY033859 CY019578
